# Comparison of plants with C3 and C4 carbon fixation pathways for remediation of polycyclic aromatic hydrocarbon contaminated soils

**DOI:** 10.1038/s41598-018-20317-0

**Published:** 2018-02-01

**Authors:** Anithadevi Kenday Sivaram, Panneerselvan Logeshwaran, Suresh R. Subashchandrabose, Robin Lockington, Ravi Naidu, Mallavarapu Megharaj

**Affiliations:** 1 Global Centre for Environmental Remediation, Faculty of Science, The University of Newcastle (UoN), University Drive, Callaghan, NSW 2308 Australia; 2 Centre for Environmental Risk Assessment and Remediation (CERAR), University of South Australia, Mawson Lakes, SA 5095 Australia; 3 Cooperative Research Centre for Contamination Assessment and Remediation of the Environments, ATC Building, The University of Newcastle, University Drive, Callaghan, NSW 2308 Australia

## Abstract

The phytoremediation technique has been demonstrated to be a viable option for the remediation of polycyclic aromatic hydrocarbons (PAHs) contaminated sites. This study evaluated the potential applicability of plants with C3 and C4 carbon fixation pathways for the phytoremediation of recalcitrant high molecular weight (HMW) PAHs contaminated soil. A 60 and 120-day greenhouse study was conducted which showed higher degradation of HMW PAHs in soil grown with C4 plants when compared to C3 plants. Also, no PAHs were detected in the maize cobs, sunflower, wallaby, and Sudan grass seeds at the end of the experiment. The effect of plants in modifying the microbial community and dynamics in the rhizosphere was also examined by measuring soil biochemical properties such as dehydrogenase activity and water-soluble phenols. The results demonstrate a substantial difference in the microbial populations between planted and unplanted soils, which in turn facilitate the degradation of PAHs. To the best of our knowledge, this study for the first time evaluated the phytoremediation efficacy through the *A. cepa* cyto- and genotoxicity assay which should be considered as an integral part of all remediation experiments.

## Introduction

Polycyclic aromatic compounds are a group of organic compounds that are categorized worldwide as priority pollutants of terrestrial and aquatic ecosystems, and sediments, primarily because they can cause mutation, cancer and interfere with the reproduction of higher organisms^1^. With the great increase in PAHs contaminated areas worldwide, many diverse technologies are being developed to solve this problem^2^. Because of their cheaper cost and minimal impact on the environment with the added advantage of *in-situ* treatment, biodegradation of PAHs using plants (phytoremediation), and microbes (microbial remediation) is considered to be a promising technology to remediate PAHs in the environment^3^.

Plants degrade organic contaminants after direct uptake from the soil and subsequent metabolism within the plants through: firstly, phytodegradation or phytotransformation processes^4^, secondly, with the aid of root exudates which enhance the activity of PAHs degrading microbial communities in the rhizosphere (rhizodegradation/rhizoremediation)^5^ and finally by increasing the activity of microbial community in the soil by composting^6^. The amount of plant uptake and accumulation of contaminants varies significantly among plant species and are mainly limited by their nonpolar and hydrophobic nature and strong association with soil organic fractions^7^. Moreover, there are several variables which include the initial soil PAH concentration, soil properties, contaminant properties and environmental factors that significantly influence the uptake, accumulation and metabolism of PAHs in plants^8^.

In general, plants can be classified according to their carbon fixation pathways, like C3, C4 and CAM (Crassulacean Acid Metabolism). Of these C3 and C4 plant species are mainly employed in phytoremediation studies^9^. Moreover, C3 and C4 plants are very different in their root composition. C4 plants have higher concentrations of amino acids and organic acids in their root exudates, whereas carbohydrate concentrations are higher in C3 plants^10^. The proportion of the dominant compounds in the root exudates especially sugars and organic acids may also differ among the C3 and C4 plants depending on the type of plants^11^. Therefore, it is important to study the performance of C3 and C4 plants in the remediation of organic pollutants mainly PAHs. However, information regarding plant responses to PAHs contaminated conditions is still lacking. Therefore, selecting suitable plant candidates is a major determinant of the success of phytoremediation studies. It is an established fact that plants release as much as 40% of their photosynthates in the form of root exudates that are released into the rhizosphere^12^. These compounds are used by the microbes in the rhizosphere as carbon and energy sources and they can then co-metabolise PAHs, a process which remains the main route for the degradation of PAHs (>3 rings)^13^. Due to the production of entirely different spectra of root exudates, plant species can differ from each other in their degradation ability and also in their enhancement of microbial activity and composition in the rhizosphere^14^. Therefore, plant species with the maximum root surface, tolerance to PAHs and ideally native to the contaminated environment, should be considered as the ideal candidates for use in the phytoremediation of PAHs^15^.

In most phytoremediation techniques, success was monitored only by the amounts of parent compounds removed while their metabolites were largely ignored. These metabolites are primarily responsible for the toxicity seen in biological organisms. Therefore, it is important to ensure that the contaminants are suitably detoxified so that they pose no risk to living organisms. In order to test the efficacy of remediation techniques, suitable toxicity assays have to be employed before declaring that the soil has been decontaminated^16^. Though various ecotoxicological assays are used to check the toxicity of the contaminants that are present in the environments, the use of higher plants like *Allium cepa* was recognized as an excellent model to detect compounds interfering with DNA replication and chromosome segregation, that occur in the environment^17^. Also*, A. cepa* root cells are also used in the single cell gel electrophoresis (SCGE) system, often referred to as the comet assay. This is regarded as a simple, cost-effective and sensitive tool for examining the genotoxicity of organic pollutants present in the environment^18^.

The purpose of this study was to determine the efficiency of plants in removing PAHs from PAHs contaminated soil. The variations in accumulation and degradation of PAHs between the C3 and C4 plants at two different growth stages (60 and 120 days) were studied in terms of the relationship with the bioavailable PAHs, while growing in field contaminated soil. Several phytoremediation reports have lacked evidence on the efficiency of phytoremediation treatments. In this study, the efficacy of phytoremediation was evaluated using cytogenetic and genotoxicity assays with *A. cepa* as the remediation endpoint assessment.

## Results

### Soil characteristics

The soil with PAHs contamination was alkaline (pH 8.5) and EC − 322 µSm^−1^ with 9% soil moisture and 52% water holding capacity (WHC). The sand, silt, and clay of the soil were 59.1%, 27.2%, and 13.6%, respectively. The dissolved organic carbon was 10 mg kg^−1^. The nitrogen and carbon were 0.04% and 2.6%, respectively. The total concentration of PAHs was 995.1 mg kg^−1^ soil. The anionic, inorganic and PAHs concentrations were summarised in Supplementary Tables S1, S2 and S3.

### Effect and impact of C3 and C4 plants on the removal of PAHs

**Figure 1:**
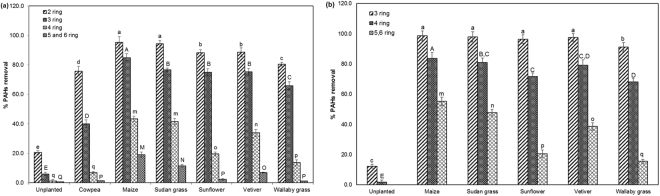
The effect of C3 and C4 plant species on the percentage removal of PAHs from the contaminated soils: (**a**) Percentage PAHs removal at 60^th^ day (**b**) Percentage PAHs removal at 120^th^ day. Bars with same letters do not differ significantly at the 5% level of significance according to Duncan Multiple Range Test (DMRT).

The 16 PAHs extracted from the contaminated soils were categorized as low molecular weight PAHs (LMW-PAHs) with two or three benzene rings; and high molecular weight PAHs (HMW-PAHs) with four or more benzene rings. The per cent removal of LMW and HMW-PAHs from the contaminated soil planted with C3 (cowpea, sunflower and wallaby grass) and C4 (maize, Sudan grass and vetiver) plant species at 60^th^ and 120^th^ day and in unplanted soils are shown in Fig. 1a and b. The presence of plant species enhanced the removal of PAHs in contaminated soils when compared to the unplanted control soils. However, the extent of PAHs removal varied with the type of plant species and molecular weight of PAHs. At the end of 120^th^ day, complete removal of 2-ringed PAHs such as naphthalene, acenaphthylene, acenaphthene, and fluorene were recorded in all the planted treatments while in unplanted control only 34.8% of 2-ringed PAHs were removed. Among the C4 plant species tested, a significantly higher amount of PAHs removal was recorded in maize for both the LMW- and HMW-PAHs at 60^th^ and 120^th^ day, on the other hand, for the C3 plants, sunflower showed the highest PAHs removal. The per cent removal of LMW-PAHs by sunflower (C3) was on a par with vetiver (C4), but for the HMW PAHs, sunflower’s per cent removal was comparatively less than that of all the C4 plant species tested. Overall, the removal of HMW PAHs from the contaminated soil by C4 plants was significantly better than the C3 plant species. The total PAHs removed by plant species tested was in the following order: maize > Sudan grass > vetiver > sunflower > wallaby grass. The per cent removal of PAHs in unplanted treatments was negligible when compared to the planted treatment, which confirms the success in the phytoremediation of PAHs.

### Influence of plant accumulation factors and bioavailability in PAHs removal

Accumulation of PAHs in maize cobs and seeds of Sudan grass, wallaby grass and sunflower were below the limit of detection. A significant variation in the effects of root/shoot concentration factors (RCF/SCF) and translocation factors (TF) on PAHs removal by the C3 and C4 plant species was observed for both the experimental period (Table 1). At 60^th^ day, in C4 plant species, except for the RCF, the other two parameters showed insignificant effects on the percentage removal of PAHs. At the end of 120 days, the SCF had no significant impact on the removal of PAHs, which was evident in all plant species tested except for the Sudan grass and vetiver. Likewise, the interaction between the RCF vs. SCF and RCF vs. TF highlighted a significant effect on the PAHs removal per cent from the soil. In Sudan grass and Vetiver, a significant interaction was observed in root concentrations, shoot concentration as well as with translocation factor at the end of 120 days. Among the C3 plants, sunflower and wallaby grass, had shown significant two way interaction between the RCF and SCF at 120 days. Furthermore, in maize, except for SCF, the interaction of all the parameters was observed to have a significant effect on PAHs removal per cent. Nevertheless, cowpea (C3) exhibited an insignificant effect in the removal of PAHs when both individual and interaction factors were considered.

**Table 1:** Multifactorial ANOVA displaying *F* and *P* values of the effects of RCF, SCF and TF individually and in combination on percentage removal of PAHs by C3 and C4 plants after 60 days of experiment.

Term	Cowpea (C3) *F value*		Sunflower (C3) *F value*		Wallaby grass (C3) *F value*		Maize (C4) *F value*		Sudan grass (C4) *F value*		Vetiver (C4) *F value*	
	60^th^ day	120^th^ day	60^th^ day	120^th^ day	60^th^ day	120^th^ days	60^th^ day	120^th^ day	60^th^ day	120^th^ day	60^th^ day	120^th^ day
RCF	0.1	n.a	9.1**	5.7*	7.7**	20.7**	24.0**	24.9**	12.0**	19.3**	9.3**	38.2**
SCF	0.0	n.a	3.5	2.4	0.4	0.0	5.4	2.2	2.6	8.1**	2.0	12.7**
TF	1.5	n.a	4.3	3.7	0.0	3.0	4.7	9.9**	3.3	12.3**	0.2	11.1**
RCF*SCF	0.2	n.a	8.0**	7.6*	4.3*	11.3**	9.3**	31.7**	8.1**	28.0**	5.0*	14.6**
RCF*TF	0.0	n.a	7.7**	0.0	6.1*	4.0	26.7**	24.5**	10.7**	18.2**	4.0*	11.5**
SCF*TF	0.0	n.a	0.5	2.8	3.3	0.1	3.5	6.56	1.6	7.0**	0.2	6.2*

*P ≤ 0.05; **P ≤ 0.01, RCF = root concentration factor, SCF = shoot concentration factor, TF = translocation factor.

### Correlation of HPCD extractability with biodegradation of PAHs by C3 and C4 plants

The HPCD extraction of the 16 PAHs from contaminated soils was compared with the percentage of PAHs removed by both C3 and C4 plants (Fig. S1a and b). A strong correlation was observed between mean values of percentage of PAHs removal and HPCD extractable fraction in both C3 and C4 plants in both the experimental durations. The relationship was in the following order: at 60 days, Sudan grass (*r*^2^ = 0.95) > maize (*r*^2^ = 0.94) > vetiver (*r*^2^ = 0.93) > sunflower (*r*^2^ = 0.92) > wallaby grass (*r*^2^ = 0.86) > cowpea (*r*^2^ = 0.77) at *P* ≤ 0.05. Whereas, after 120 days of experimental period the relationship was in the order of maize (*r*^2^ = 0.95) > Sudan grass (*r*^2^ = 0.92) > vetiver (*r*^2^ = 0.94) > sunflower (*r*^2^ = 0.89) > wallaby grass (*r*^*2*^ = 0.89) at *P* ≤ 0.05. These results revealed that HPCD extraction reasonably predicted the bioavailable concentration to the plants in the PAHs contaminated soils.

### Changes in soil enzymatic activity

The changes in soil dehydrogenase activity (DHA) and water-soluble phenols as influenced by C3 and C4 plants after 60^th^ and 120^th^ days are summarised in Table 2. The DHA was measured at the end of the experimental period in unplanted control and rhizosphere (region surrounding the root) soil samples in order to estimate the influence of plant species on soil microbial activity. After 60 days, the DHA in the planted soils was increased from 4–18 times the original amount when compared to the unplanted soils. Similarly, at the end of 120 days, a huge variation in DHA was observed in the unplanted and planted soils with a 9-fold to 47-fold increase in the planted treatments compared to the unplanted control soils. The DHA was higher in soil planted with C4 plant species than the C3 plants. Among all the planted treatments, the highest DHA was recorded in maize at the end of 60 days (5.35 µg of TPF g^−1^ of soil h^−1^) and 120 days (19.32 µg of TPF g^−1^ of soil h^−1^) while the lowest in the rhizosphere soil of cowpea (1.07 µg of TPF g^−1^ of soil h^−1^). The water-soluble phenols (WSP) were estimated and expressed in terms of vanillic acid equivalents. The WSP concentrations followed a similar trend as that of DHA. The WSP in the planted soils ranged between 2.46–4.96 µg and 1.3–14.5 µg vanillic acid (VA) g^−1^ of soil h^−1^ at 60 and 120^th^ days, respectively. The soil remediated with maize (C4) showed a 3-fold increase with the WSP concentration from 60^th^ to 120^th^ day. On the other hand, a minimal increase in WSP was observed in the soils planted with C3 plants.

**Table 2:** Changes in soil enzymatic activities at 60^th^ and 120^th^ day of phytoremediation.

Treatments	DHA (µg of TPF g^−1^ of soil h^−1^)		WSP (µg VA g^−1^ of soil)	
	60^th^ day	120^th^ day	60^th^ day	120^th^ day
0^th^ day	0.08 ± 0.01^g^	0.08 ± 0.01^f^	0.56 ± 0.03^g^	0.56 ± 0.03^g^
Unplanted	0.29 ± 0.02^f^	0.41 ± 0.04^e^	1.14 ± 0.21^f^	1.35 ± 0.09^e^
Cowpea	1.07 ± 0.12^e^	n.a	2.46 ± 0.07^e^	n.a
Maize	5.35 ± 0.24^a^	19.32 ± 0.80^a^	4.96 ± 0.09^a^	14.46 ± 0.89^a^
Sudan grass	4.08 ± 0.18^b^	15.79 ± 0.44^b^	4.32 ± 0.13^b^	10.87 ± 0.50^b^
Sunflower	2.73 ± 0.05^d^	5.97 ± 0.11^cd^	3.11 ± 0.05^c^	5.32 ± 0.16^c^
Vetiver	2.94 ± 0.16^c^	8.18 ± 0.68^c^	3.21 ± 0.09^c^	6.38 ± 0.09^c^
Wallaby grass	2.60 ± 0.11^d^	3.56 ± 0.34^d^	2.91 ± 0.09^d^	3.34 ± 0.07^d^

Values are means and standard deviation of triplicate measurements. Means sharing same letters in the superscript position do not differ significantly at the 5% level of significance according to DMRT.

### Cyto- genotoxicity using *A. cepa* bioassay

**Figure 2:**
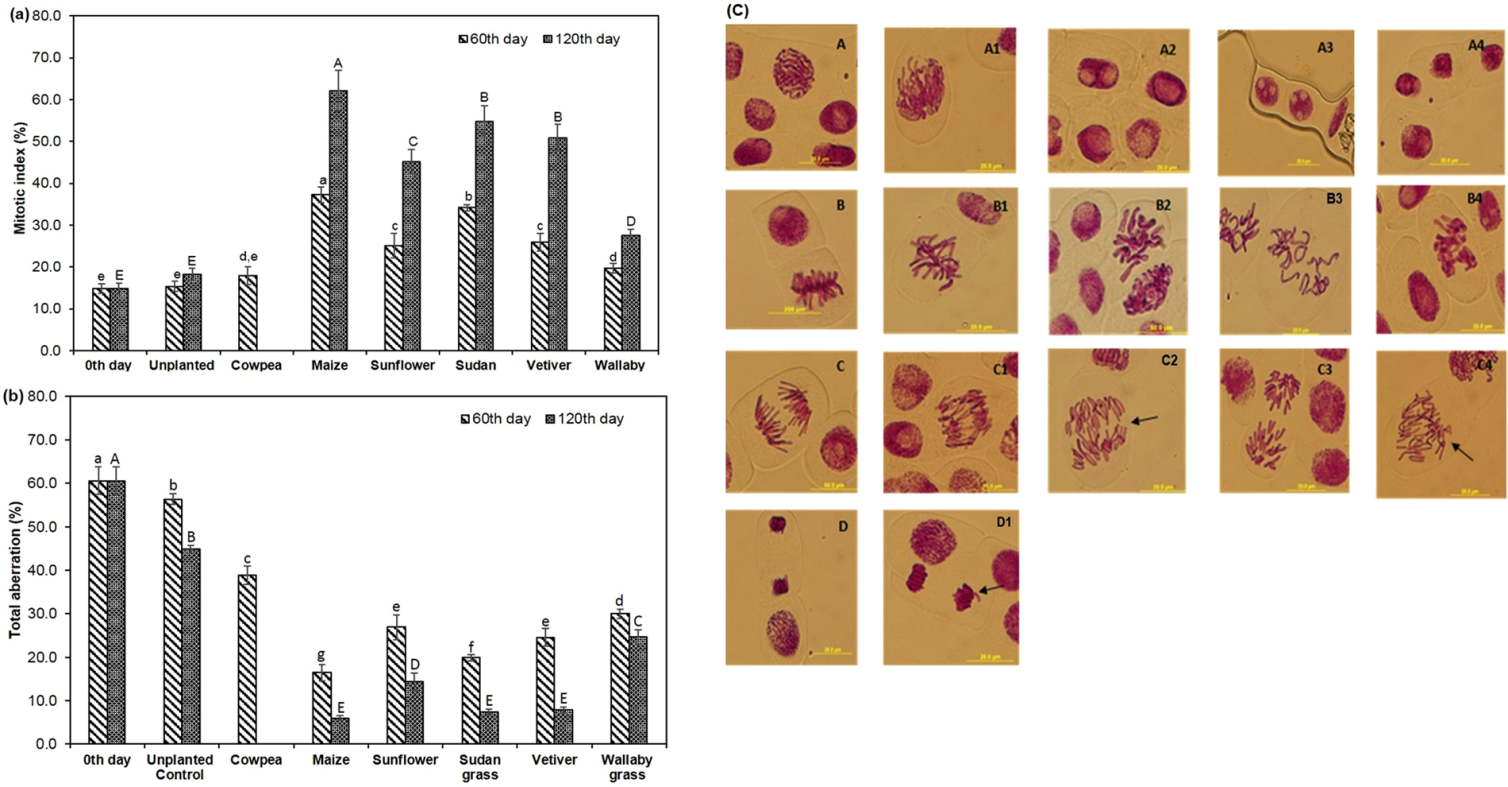
**(a)** Remediation efficacy testing with *A. cepa* meristem root cells exposed to unplanted contaminated and phytoremediated soils (60^th^ and 120^th^ day) - Mitotic index (%); **(b)** Remediation efficacy testing with *A.cepa* meristem root cells exposed to unplanted contaminated and phytoremediated soils (60^th^ and 120^th^ day) - total aberration (%) Bars with same letters do not differ significantly at the 5% level of significance according to Duncan Multiple Range Test (DMRT); **(c)** Chromosomal aberrant cells observed in *A. cepa* grown in contaminated soils (A) Prophase, (B) Metaphase, (C) Anaphase, (D) Telophase, (A1) Disturbed prophase, (A2) Bivacuolated cells, (A3) Multivacuolar cells, (A4) Micronuclei, (B1) Fragments, (B2) C-mitosis, and bridges, (B3) Disturbed metaphase, (B4) Sticky metaphase, (C1) Anaphase bridges, (C2) Laggards, (C3) Multipolar anaphase, (C4) Anaphase with vagrant laggards, (D1) Anaphase fragments.

The phytoremediated soil both after 60^th^ and 120^th^ day was planted with *Allium cepa* to analyze remediation efficacy by evaluating the reduction in genotoxic effects. The results were compared with those obtained with unplanted control soil. An improvement in mitotic activity was observed after remediating PAH-contaminated soils with plant species (Fig. 2a). The mitotic index (MI) (%) of meristem onion cells grown in the 0^th^ day contaminated soil was significantly lower (14.9%) compared to all the phytoremediated soils. There was no significant difference in MI values between the unplanted control and contaminated soils before remediation. The remediated soil at the end of 120 days, showed an increase in MI value when compared to 60^th^ day remediated soil in all planted treatments. Of all the remediation treatments with plants, at the end of 120 days, a four-fold increase in MI was observed in maize rhizosphere soils (62.1%), when compared to the contaminated soil prior to remediation. The cytotoxic effect of the remediated soils from planted treatments was significantly reduced compared to 0^th^-day control and unplanted contaminated soils. However, the aberrant chromosomes were observed in all the treatments, and the highest aberration percentage was recorded in contaminated soil grown with cowpea (60^th^ day) (Fig. 2b) The chromosomal aberrations that were observed in the different stages of cell cycle includes bridges, breaks, micronuclei, laggards, C-mitosis, disrupted metaphase and sticky chromosome. The chromosomal aberrations like laggards, micronuclei, and vagrants were observed in cowpea planted contaminated soils (Fig. 2c). A large number of sticky chromosomes, disrupted metaphase and C-mitosis aberrations were observed in the contaminated soil before phytoremediation and in unplanted control soils. The total chromosomal aberration percentage and the percentage of PAHs removed from the soil by the plant species, indicated a strong negative correlation at *P* ≤ 0.01 with *r*^*2*^ = −0.87 (60^th^ day) and *r*^*2*^ = −0.91 (120^th^ day)). In contrast, the mitotic index and percentage of PAHs removed showed a strong positive correlation *r*^*2*^ = 0.97 (60^th^ day) and *r*^*2*^ = 0.98 (120^th^ day) at *P* ≤ 0.01.

**Figure 3:**
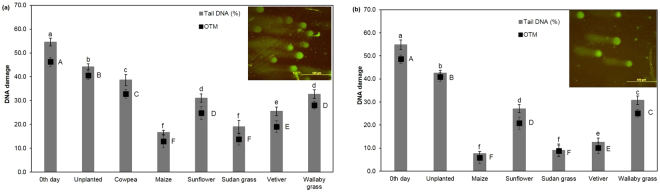
**(a**) Levels of genotoxicity seen in *A. cepa* meristem root cells exposed to unplanted contaminated and phytoremediated soils (60^th^ day); **(b)** Levels of genotoxicity seen in *A. cepa* meristem root cells exposed to unplanted contaminated and phytoremediated soils (120^th^ day). The means sharing same capital letters do not differ significantly at the 5% level of significance according to DMRT for TD (%) and OTM (%).

The DNA damage in onion meristematic cells was determined using the comet assay. The percentage tail DNA (%TD) and olive tail moment (OTM) were used to express the genotoxicity in onion root meristem cells (Fig. 3a,b). A significantly large amount of tail damage with higher 43.8% TD and 36.9% OTM was found in the onion roots grown in 0^th^ day contaminated soil and in the unplanted soil when compared with phytoremediated soils. The % TD and OTM in phytoremediated soils varied from 37.1–15.9% and 26.8–10.4% respectively. The correlation analysis indicated a strong positive relationship between the PAHs levels in the soils and onion root meristem DNA damage parameters like OTM and TD (%) *r*^2^ = 0.87 and 0.92, respectively.

## Discussion

The present study compared the potential of C3 and C4 plant species to remediate PAHs contaminated soils. The amount of PAHs removed was observed to be higher in the planted treatments than in the unplanted control. This suggested that the plant’s presence in the contaminated soil significantly enhanced the removal of PAHs. In general, the extent of PAHs removed was reported to decrease when the molecular weight and ring number of PAHs increased^19^. Likewise, the results of the present study expand these findings, confirming that the LMW-PAHs (2 and 3-ringed) were degraded efficiently by the plants when compared to HMW-PAHs (4, 5 and 6-ringed). This differences in degradation of LMW and HMW - PAHs were probably due to the recalcitrant nature of the HMW - PAHs, and thus they remain persistent in soil over time. The phytoremediation of PAHs contaminated soils not only depends on the physicochemical property of PAHs but also depends on the nature of plants as well as the biologically available concentration of PAHs^20^. Numerous methods were formulated to detect the bioavailable concentration of hydrophobic organic contaminants^21^. In our study, the HPCD extraction method was used to detect the bioavailable concentration of PAHs in the soil. Earlier studies reported on the positive correlation between the PAHs bioavailability using the HPCD method and biodegradable fraction of PAHs^22^. Similarly, in this study, a significant positive correlation was observed between the HPCD extractable bioavailable concentration and the removal of PAHs by plants. Therefore, the HPCD method used here predicted the bioavailable concentration of PAHs to a reliable extent. Indeed the percentage of PAHs removed at the 120^th^ day was higher than the HPCD predicted bioavailable PAHs fraction especially for LMW PAHs in C3 and C4, and to some extent for HMW PAHs in C4 plants. This could be due to the action of root exudates in improving the bioavailability of PAHs to the plants. Among the planted treatments, C4 plants exhibited a larger percentage of overall PAHs removal than the C3 plants (Fig. S2) which could probably due to the plant-specific rhizosphere effect. Nevertheless, the performance of C3 plants in removing the LMW weight PAHs was comparable to the C4 plants. Also, the bacteria able to utilise LMW-PAHs as carbon source are widespread in soils^2^. Of the C3 plant species, sunflower showed the highest removal percentage of LMW-PAHs, but generally, their performance in remediating HMW-PAHs was far below that of all the C4 plant species.

The uptake of the PAHs by the plants is influenced by several factors such as bioavailability, plant lipophilicity, soil organic matter, and log *Kow* value^23^. Plants uptake PAHs from the soil and subsequently accumulate in the root system or are translocated to the above-ground parts of the plants through transpiration, and some portions are metabolized by the plant tissues^24^. The variation in root accumulation of the PAHs observed here may also be due to the difference in root lipid content of the C3 and C4 plant species^25^. The RCF was observed to be significant compared to SCF which could be mainly due to the adherence of PAHs onto the root surface due to their lipophilic nature, and they may not be significantly transferred into the inner xylem because in xylem the translocation is water-based. Nevertheless, the translocation of a certain fraction of PAHs to the shoots from the roots cannot be entirely neglected^26^. This outcome predicts that the PAHs concentration in plant roots could primarily be due to adsorption by the root epidermis^27^.

The quantities of PAHs stored in other plant tissues were much smaller, which can be due to limited uptake of PAHs in plant tissues, or they could also be metabolized in the tissues to some extent, thus reducing their accumulation. While in this study the accumulation of PAHs in the plant shoots was low, and the SCF showed a positive correlation with the percentage of PAHs removed, mainly in the C4 plants. This may be explained by the higher transpiration rate of C4 plants leading to the translocation of PAHs from roots to the shoots than their C3 counterparts. Further studies on the constituents of PAHs in xylem and phloem sap may determine their translocation rates. Even though the translocation of PAHs from root to shoot occurs at a low rate, the concentration of PAHs in shoots and their corresponding translocation concentrations were observed to have an insignificant effect on the removal of PAHs in all the plant species except for Sudan grass and vetiver. Nevertheless, PAHs were not detected in the plant parts such as maize cobs, and seeds of sunflower, Sudan grass and wallaby grass. This provides positive support for the phytoremediation of PAHs in which the contaminants were degraded mainly by the presence of PAH-degrading microbial population in the rhizosphere. This overcomes criticisms made about the phytoremediation technique, that it may transfer the contaminants to higher organisms through the food chain.

Under stressful conditions plants up-regulate the shikimate and acetate pathways by producing larger amounts of phenolic compounds as root exudates^28^. Due to the structural similarity of some components of root exudates with the contaminants, root exudates aid in the co-metabolism of contaminants, thus leading to the proliferation of the microbial population in the rhizosphere regions^29^. The soil dehydrogenase activity is often correlated with the size of active microbial populations in soil, and the water-soluble phenols in the PAHs contaminated soils are derived from two sources: one is by the degradative intermediates of PAHs by ring cleavage, and other from the plant-produced root exudates^30^. In this study, higher dehydrogenase activity and water-soluble phenols were recorded in the planted soil. This could probably be due to the root exudates produced by the plants promoting the proliferation of the microbial population in the rhizosphere^31^. A strong positive correlation was observed between the percentage of PAHs removal with dehydrogenase and water-soluble phenolic content. This finding agrees with those of previous studies on the relationship between water-soluble phenols, and the amount of PAHs removed from soil^32^. Results indicate the efficiency of plant species in enhancing WSP content and activity of dehydrogenase in the soil and thereby enhancing rhizodegradation of PAHs.

The C4 plants showed a higher concentration of the water-soluble phenols and dehydrogenase activity than the C3 plants. This is in keeping with the fact that, the C4 plants have higher photosynthetic rate compared to C3 plants which promoted the enhanced production of plant photosynthates^33^. The photosynthates that are produced by plants subsequently translocated from shoot to roots as rhizodeposits or root exudates. The root exudates contain soluble organic carbon content and those plants which have the capacity to release significant amounts of phenols will selectively increase the microbial population able to degrade PAHs in the rhizosphere region^34^. The enhanced tolerance of plants to stressful environment mainly depends on the accumulation of higher plant biomass, which is higher in case of plants with C4 photosystems. In addition, C4 plants with the enhanced nitrogen and water use efficiency have a competitive advantage over the C3 plants^35^. It can, therefore, be concluded that plants especially those with a C4 photosynthetic pathway enhanced the degradation of soil PAHs by stimulating the microbial population in the rhizosphere region.

The cytogenetic assays were performed using *A. cepa* to verify the efficiency of the remediation strategy, since PAHs both as parent compound or metabolites may impose genetic damage in living organisms, which can lead to inheritable genetic alterations^36^. The *A. cepa* test is the most sensitive one and produces reliable results, which are extensively used for the risk assessment of emerging contaminants^37^. The levels of cytogenetic damage and genotoxicity were estimated by observing cytological parameters such as the mitotic index and number of chromosomal abnormalities. The mitotic index is often used to check the cytotoxicity of a substance^38^. In this study, the mitotic index (%) of the phytoremediated soils (at 60^th^ and 120^th^ day), increased when compared to 0^th^ day and unplanted control. This shows that PAHs contaminated soil before remediation has high cytotoxicity and this interferes with the cell division by prolonging the S phase and inhibiting DNA and protein synthesis^39^. The pronounced chromosomal abnormalities observed in the soil before phytoremediation confirm that PAHs contaminated soils exerted both eugenic and clastogenic effects before the phytoremediation. The most frequent abnormalities in the unplanted control and 0^th^-day soil were anaphase bridges, micronuclei, and stickiness. The chromosome bridges leading to a chromatid break indicated the clastogenic effects of PAHs in plants. The micronuclei were formed as a result of damage in the chromosomes such as breaks and losses that were incorrectly repaired by the parent cells^36^. Therefore, the induction of the micronuclei in this study suggests that PAHs contaminated soils have a strong clastogenic effect by inhibiting the spindle fibers^40^. Vagrant chromosomes and C-mitosis observed in the 0^th^-day soil may increase the risk of aneuploidy as reported by Leme and Marin-Morales^38^. A pronounced stickiness observed in a typical metaphase and anaphase in the 0^th^ day and unplanted control soil was often related to the severe toxic effect of chromosomes probably leading to cell death^41^. Nevertheless, in this study, the chromosome stickiness was not detected in the maize, sunflower, Sudan grass and vetiver planted soils, which confirms the efficacy of phytoremediation in removing toxic compounds.

Previously, the application of comet assay technique with higher plants was often limited, because of their intact solid tissue and extremely thicker cell wall, which requires pre-treatments to free nuclei from the cells. However, the technology was made easier with the advancement of rapid cell digestion pre-treatments, and many higher plants, especially *A. cepa* was employed in the identification of genotoxic substances because of their high reliability and lack of false negatives^42^. In the present study, tail length and olive tail moment were used to analyze the efficacy of the phytoremediation treatments. Significantly, more DNA damage was detected in the unplanted control soil when compared to the phytoremediated soils. This was mainly due to the higher concentration of PAHs in unplanted soil compared to phytoremediated soils. A firmly positive correlation was found between the olive tail movement and concentration of PAHs in the soil. This clearly indicates that the plants detoxified the cytotoxic effects of PAHs in the contaminated soil. This finding demonstrates the suitability and greater sensitivity of *A. cepa* comet assay in detecting ecotoxicological substances in the environment.

## Conclusion

This study explored the potential of C3 and C4 plant species to remediate PAHs contaminated soils. The plant species used have great potential in the remediation of PAHs contaminated soils. It emerged that C4 plants can significantly enhance the removal of PAHs, especially high molecular weight PAHs such as 4, 5 and 6 ringed PAHs when compared to C3 plants, which is probably due to their greater ability to accelerate the microbial community in the rhizosphere region. This study also demonstrates the usefulness of *A. cepa* cytogenetic assay in testing the remediation efficacy of PAHs contaminated soils following phytoremediation. This study lays the foundation for knowing the performance of plants with C3 and C4 photosystems in PAHs contaminated soil.

## Methods

### Chemicals and solutions

Sixteen PAHs mix standards (EPA TCL Polynuclear Aromatic Hydrocarbons mix) were purchased from Supelco (Sydney, Australia). All the other solvents and the chemicals used in the study were analytical grades purchased from Sigma-Aldrich (Sydney, Australia).

### Soil characterisation

The soil sample (PAHs contaminated) was collected from a landfill site in Dublin, South Australia. Prior to further analysis, the soils were air dried and sieved (<2 mm). Using probes (Smart CHEM-pH, TPS), the pH and EC of the soils were measured in soil/water (Millipore 18.0 M.Ω cm) suspensions (1: 5 ratios). Dissolved organic carbon (DOC) was determined from the filtered (0.45 µm) supernatant resulting from an overnight end-over-end shaking (Southern cross scientific, Australia) and centrifugation at 4000 × g for 20 min. The same filtrate was used for TOC analysis (Shimadzu TOC- LCSH). Ion chromatography (Dionex ICS-2000) with AS19 column was used for the estimation of inorganic anions such as (F^−^, Cl^−^, Br^−^, NO_2_^−^, NO_3_^−^, SO_4_^2−^, and PO_4_^3−^).

The combustion method was carried out to measure the total carbon and nitrogen using Leco induction furnace (LECO: TruMac, CN/S analyzer). A 0.5 g of the soil sample in duplicates along with certified reference soil (Montana Soil SRM 2711) and blank were digested in *aqua regia* (1 HNO_3_:3 HCl)^43^ using MARS 5 microwave digestion system (HP 500, CHEM). The digests were cooled to room temperature and filtered (0.45 µm Millipore™ filters) and the total concentrations of P, K, S, Cr, Mn, Ni, Co, Cu, Zn, As, Cd and Pb were determined by USEPA method 3051 H by Inductively Coupled Plasma-Mass Spectroscopy (ICP-MS) (Agilent 7500c). The hydrometer method^44^ was used for the determination of sand, silt and clay contents. The water holding capacity (WHC) was determined following the method by Grant^45^.

### Greenhouse experiment and conditions

A preliminary screening test was conducted for 50 days before this experiment with 14 different plant species comprising nine plants with C3 and five plants with a C4 photosynthetic pathway to check the ability of plant species to withstand and degrade PAHs in contaminated soils^46^. Based on the performance of plant species in terms of percentage PAHs removal, biochemical and physiological parameters, six best performing plants comprising three C3 plants (cowpea, sunflower and wallaby grass) and three C4 plants (maize, Sudan grass and vetiver) were chosen to test for their potential applicability in the phytoremediation of field contaminated soils (Supplementary Table S4)^46^. The seeds were surface sterilized by rinsing with 95% ethanol for 10 seconds and then with 10% hydrogen peroxide (H_2_O_2_) and 1.25% sodium hypochlorite (NaOCl_2_) for 3–5 min followed by rinsing with sterile water. Depending on size, 5 or 10 seeds were planted (at 5 mm depth) in polypropylene pots containing 1 kg of soil for 60 and pots containing 4.5 kg soil for 120 days experimental period. Unplanted control pots containing only soil were also maintained. Planted treatments and unplanted control were maintained with three replicates and randomly arranged. The day of sowing was considered to be the 0^th^ day. Thinning was carried out after 10 days of germination leaving two seedlings per pot for 60 days and leaving one plant per pot for maize and sunflower and 2 plants per pot for wallaby and Sudan grass for 120 days experiment. For vetiver, one ~100 mm slip (with 25 mm roots) was planted at 50 mm depth per pot. Since cowpea exhibited poor performance with stunted growth and a lower rate of PAHs degradation after 60 days, cowpea plants were not included in the 120 days experimental studies. The experimental condition was a light/dark cycle of approximately 16/8 h at (25 ± 3/12 °C). This experimental condition was selected based on the published studies on the comparison of C3 and C4 plant photosynthetic pathways that were conducted in greenhouse experimental conditions^46–48^. The selected greenhouse temperature lies in the optimal temperature range of C3 and C4 plants at which their photosynthesis remains unaffected^49^.

Around 60% (w/w) soil water holding capacity was maintained throughout the duration of the experiment. For 60 day experiment, soils were amended with 10% Hoagland’s solution (15 mL/pot) at weekly intervals^50^. Whereas, for 120 day experiment, the soils along with Hoagland’s solution were fertilized with NPK mixture @ 1 g kg^−1^of soil containing N:P_2_O_5_:K_2_O in the ratio of 1:0.35:0.8. At the end of 60^th^ and 120^th^ day, the rhizosphere soils were collected as described by Szmigielska *et al*.^51^ and divided into two sets, one set was used for determination of dehydrogenase activity and water-soluble phenols, and the other set was used for the analysis of PAHs (stored at −18 °C). All the plant species were harvested at the end of 60^th^ and 120^th^-day experimental period. The collected plant samples were freeze-dried and homogenized with liquid nitrogen and used for the PAHs analysis.

### Soil enzyme analysis

Dehydrogenase activity (DHA) was analyzed in terms of TTC (2,3,5-triphenyl tetrazolium chloride) reduction to a colored product known as triphenyl formazan (TPF)^52^, which was then analyzed at 485 nm in a microplate reader (Bio-Tek^®^SynergyTM HT equipped with KC4 software).

Water-soluble phenols were quantified spectrophotometrically according to the method described by Carter^53^. Absorbance was read at 750 nm in a microplate reader (Bio-Tek^®^SynergyTM HT equipped with KC4 software). The amount of phenolic compounds is expressed as vanillic acid equivalents (µg vanillic acid g^−1^ soil).

### Extraction and analysis of PAHs from soil and plants

The initial concentration of PAHs was determined by collecting 20 g of the soil and stored at −18 °C until analysis. The extraction of PAHs was done according to Huang, *et al*.^54^. At the end of the experimental period (60^th^ and 120^th^ day), the PAHs accumulation in each plant sample was determined as suggested by Gao, *et al*.^55^. High-performance liquid chromatography (HPLC) was used to analyze the extracted samples as described earlier^46^. Zorbax Eclipse Column XDB - C18 (Agilent Technologies 1200) was used for the separation. Methanol and water (30:70) was used as the mobile phase during first 5 min and the ratio was increased gradually to 100:0 during the next 10 min and maintained for 5 min with the total run time was 25 min including a post-run of 5 min. Ten µL was used as injection volume. The UV-Vis detector at 254 nm and the UV-FLD detector with excitation of 297 nm and emission of 405 nm were used for detection. A certified standard mixture of PAHs (TCL Polynuclear Aromatic Hydrocarbons Mix-Ref 4-8905, Supelco, Bellefonte, PA, USA) was used for external calibration. A solvent blank and known standard were injected after every ten samples in order to check the instrument’s sensitivity and also to make sure that the column was clean without traces of PAHs that are carried over between samples. The soil extracts, roots, and shoots were analysed for concentrations of PAHs and calculated on dry weight basis. The PAHs removal percentage was calculated with the following equation:

1 $${\rm{Removal}}\,( \% )=\frac{100\ast (CI-CT)}{CI}.$$

Where,

CI was the initial PAHs concentration present in the soil, and

CT was the final concentration of PAHs after remediation by plant species.

RCF is calculated as contaminant concentration in roots and their respective soil concentration, whereas, SCF is calculated as contaminant concentration in shoots and their individual soil concentration. TF is calculated in terms of SCF to its corresponding RCF^56^.

### Bioavailability – Hydroxypropyl-β- cyclodextrin (HPCD) extraction method

The bioavailability of hydrocarbons was measured using aqueous Hydroxypropyl-β- cyclodextrin (HPCD) extraction. The extraction was adapted from the methods described by Oleszczuk^57^. Accordingly, 1.5 g of soil in triplicates (dried and sieved at 2 mm) placed in 40 mL Teflon centrifuge tubes were mixed with 20 mL of 50 mM aqueous solution of HPCD for all samples along with an analytical blank. The tubes were sealed and placed on an orbital shaker (Thermo Fisher Scientific, Australia) at 100 rpm for 24 h in the dark followed by centrifugation at 4,000 g for 30 min (Beckman JA21/2 Centrifuge, USA). The supernatant was then discarded. The residual PAHs were measured by ultrasonic solvent extraction method as described above. The bioavailable fraction of PAHs was then calculated as the difference between the total and residual PAHs after HPCD extraction.

### Cytotoxicity and genotoxicity bioassay with *Allium cepa*

The phytoremediated soils (60^th^ and 120^th^ day) and unplanted soil were assessed for cytogenetic and genotoxicity using the *A. cepa* bioassay at the end of the experiment. The seeds of *A. cepa* were surface sterilized as described earlier. Ten sterilized seeds (per pot) were then allowed to germinate in 200 g of the soils that have undergone phytoremediation in addition to unplanted soil and also the soil prior to remediation (0^th^-day control soil) in polypropylene containers in duplicates in the greenhouse for ten days. The roots were harvested when they were about 1.5–2.0 cm long during their second mitotic cycle and were used for cytological analysis.

The roots were analyzed for their cyto**-** and genotoxicity following Feulgen’s squash technique. Accordingly, the harvested roots were fixed immediately in the mixture of absolute ethanol and acetic acid (3:1 ratio) (Carnoy’s reagent) for 24 h at 4 °C. The roots were transferred to the tube containing distilled water. After that, the roots were hydrolyzed with 1 N HCl in a water bath at 60 °C for 10 min. The hydrolyzed roots were then washed with distilled water and transferred to a tube containing 70% (v/v) ethanol and stored at 4 °C until further use. The roots were then stained with basic Fuchsin dye and kept in the dark for 2 h. About 2 mm stained root tips were squashed with 45% of glacial acetic acid using coverslips and observed for any chromosomal changes using an Olympus BX41 epifluorescent microscope at 10× and 100× magnifications. The microscopic analysis included recording the mitotic index, a number of micronuclei in the interphase cells, and aberrant cells during metaphase, anaphase, and telophase. The mitotic index was calculated as the number of the dividing cells per number of 500 observed cells per slide for each treatment and control. Five slides per sample were analyzed. Chromosomal aberrations at each stage of the division were scored for each remediation treatment^41^.

### Extraction of *A. cepa* root meristem cell nuclei and comet assay

The *A. cepa* meristematic root tips (5 mm) were excised, and about 10 mg of root tips were chopped finely with sterile scalpel in 500 µl of ice-cold nuclei isolation buffer containing 0.2 M Tris-HCl (pH 7.5), 4 mM MgCl_2_.6H_2_O and 0.5% w/v Triton X-100 and 4 µg 4′,6-diamidino-2-phenylindole (DAPI)^58^. The suspension was filtered through nylon mesh (50 µm) followed by centrifugation at 200 g for 5 min (4 °C). The pellet was resuspended in 100 µl of Tris-HCl buffer (pH 7.5). The integrity of the nuclei was determined by staining the nuclei with ethidium bromide (10 µg mL-1) and observed under a microscope (100× magnification)^59^.

DNA damage and tail movements were evaluated by the use of alkaline single cell gel electrophoresis or comet assay, according to Singh, *et al*.^60^ methods. Fifty microlitres of the nuclei suspension were mixed with 150 µL of low melting agarose and mixed thoroughly by pipetting. Comet assay slides were coated with 50 µL of cells-agarose suspension and allowed it to solidify at 4 °C for five mins. The alkaline comet assay was performed according to the manufacturer’s instructions (Trevigen comet assay protocol, 8405 Helgerman Ct.). At the end of the assay, the slides were analyzed using a fluorescence microscope (Olympus BX41) at 10× magnification. DNA damage was expressed as the tail DNA and olive tail moment (OTM) using an image analysis computerized method by CometScore software (TriTek Corp., USA). The olive tail movement was measured as the distance between the center of gravity of head and tail.

### Statistical analysis

All the statistical analyses were done using IBM SPSS, PASW statistical software version 21.0 and Minitab 17 statistical software package. Analysis of Variance (ANOVA) determined the overall treatment effects, and when necessary, the data were log transferred to meet the ANOVA. Tukey’s multiple comparison tests were used to group the plant species tested based on the percentage of PAHs removed from the soil on the level of significance at *P* ≤ 0.05. The impact of the root, shoot concentrations, and translocation factors on the PAHs removal were estimated using multifactorial ANOVA. The normal distribution of the response and per cent PAHs removal by C3 and C4 plant species were analyzed using normal probability plot. It showed that all of the data points were close to the line with no outlier and therefore all the data points were used for the analysis. Percentage HPCD extractable fraction was correlated with the percentage of PAHs removed from the soil by plants using linear regression. In order to study the significant correlation among the different parameters tested on the remediation of PAHs, a correlation coefficient matrix was done using Pearson’s method. Duncan’s multiple range test (DMRT) was used for three things: grouping PAHs removed by plants and for cytogenetic assay and the comet assay. A dendrogram was constructed with centroid linkage and Euclidean distance, based on the performance of different plants used in the study.

## Electronic supplementary material

Supplementary Information

## References

[1] ATDSR (ed. Public Health Service US Department of Health and Human Services, Agency for Toxic Substances and Disease Registry) (USA, 2005).

[2] Kuppusamy S (2017). Remediation approaches for polycyclic aromatic hydrocarbons (PAHs) contaminated soils: Technological constraints, emerging trends and future directions. Chemosphere.

[3] Macek T, Mackova M, Káš J (2000). Exploitation of plants for the removal of organics in environmental remediation. Biotechnol. Adv..

[4] Edwards, R., Dixon, D. P., Cummins, I., Brazier-Hicks, M. & Skipsey, M. In *Organic Xenobiotics and Plants* 125–148 (Springer, 2011).

[5] Corgié S, Joner EJ, Leyval C (2003). Rhizospheric degradation of phenanthrene is a function of proximity to roots. Plant Soil..

[6] Barker AV, Bryson GM (2002). Bioremediation of heavy metals and organic toxicants by composting. Sci. World J..

[7] Kim IS, Park J-S, Kim K-W (2001). Enhanced biodegradation of polycyclic aromatic hydrocarbons using nonionic surfactants in soil slurry. Appl. Geochem..

[8] Kapusta P, Szarek-Lukaszewska G, Kiszka J (2004). Spatial analysis of lichen species richness in a disturbed ecosystem (Niepolomice Forest, S Poland). The lichenologist.

[9] Srivastava, J., Kalra, S., Chandra, H. & Nautiyal, A. Response of C3 and C4 plant systems exposed to heavy metals for phytoextraction at elevated atmospheric CO_2_ and at elevated temperature. *Environmental Contamination*. *Intech open Publisher, Croatia*, 3–16 (2012).

[10] Nabais C (2011). Effect of root age on the allocation of metals, amino acids and sugars in different cell fractions of the perennial grass *Paspalum notatum* (Bahiagrass). Plant Physiol. Bioch..

[11] Vranova V, Rejsek K, Skene KR, Janous D, Formanek P (2013). Methods of collection of plant root exudates in relation to plant metabolism and purpose: A review. J. Plant Nutr. Soil Sci..

[12] Neumann G, Römheld V (2007). The release of root exudates as affected by the plant physiological status. The Rhizosphere: Biochemistry and organic substances at the soil-plant interface.

[13] Cerniglia CE (1992). Biodegradation of polycyclic aromatic hydrocarbons. Biodegradation.

[14] Guo M (2017). The influence of root exudates of maize and soybean on polycyclic aromatic hydrocarbons degradation and soil bacterial community structure. Ecol.Eng..

[15] Aprill W, Sims RC (1990). Evaluation of the use of prairie grasses for stimulating polycyclic aromatic hydrocarbon treatment in soil. Chemosphere.

[16] Mendonca E, Picado A (2002). Ecotoxicological monitoring of remediation in a coke oven soil. Environ. Toxicol..

[17] Pakrashi S (2014). *In vivo* genotoxicity assessment of titanium dioxide nanoparticles by *Allium cepa* root tip assay at high exposure concentrations. PLoS One.

[18] de Lapuente, J. *et al*. The Comet Assay and its applications in the field of ecotoxicology: a mature tool that continues to expand its perspectives. *Front.Genet*. **6** (2015). 10.3389/fgene.2015.00180 PMC4454841 26089833

[19] Haritash A, Kaushik C (2009). Biodegradation aspects of polycyclic aromatic hydrocarbons (PAHs): a review. J. Hazard. Mater..

[20] Reid BJ, Jones KC, Semple KT (2000). Bioavailability of persistent organic pollutants in soils and sediments-a perspective on mechanisms, consequences and assessment. Environ. Pollut..

[21] Cui X, Mayer P, Gan J (2013). Methods to assess bioavailability of hydrophobic organic contaminants: Principles, operations, and limitations. Environ. Pollut..

[22] Papadopoulos A, Paton GI, Reid BJ, Semple KT (2007). Prediction of PAH biodegradation in field contaminated soils using a cyclodextrin extraction technique. J. Environ.l Monitor..

[23] Ryan J, Bell R, Davidson J, O’connor G (1988). Plant uptake of non-ionic organic chemicals from soils. Chemosphere.

[24] Trapp S, Matthies M, Scheunert I, Topp EM (1990). Modeling the bioconcentration of organic chemicals in plants. Environ. Sci.Technol..

[25] Gao Y, Zhu L (2004). Plant uptake, accumulation and translocation of phenanthrene and pyrene in soils. Chemosphere.

[26] Simonich SL, Hites RA (1995). Organic pollutant accumulation in vegetation. Environ. Sci.Technol..

[27] Tao S (2004). Polycyclic aromatic hydrocarbons (PAHs) in agricultural soil and vegetables from Tianjin. Sci. Total Environ..

[28] Siqueira JO, Nair MG, Hammerschmidt R, Safir GR, Putnam AR (1991). Significance of phenolic compounds in plant‐soil‐microbial systems. CRC Cr.Revi. Plant Sci..

[29] Nzila A (2013). Update on the cometabolism of organic pollutants by bacteria. Environ.Pollut..

[30] Sun T-R (2010). Roles of abiotic losses, microbes, plant roots, and root exudates on phytoremediation of PAHs in a barren soil. J. Hazard. Mater..

[31] Soleimani M (2010). Phytoremediation of an aged petroleum contaminated soil using endophyte infected and non-infected grasses. Chemosphere.

[32] Cheema SA (2009). Enhancement of phenanthrene and pyrene degradation in rhizosphere of tall fescue (*Festuca arundinacea*). J. Hazard. Mater..

[33] Ehleringer JR, Sage RF, Flanagan LB, Pearcy RW (1991). Climate change and the evolution of C4 photosynthesis. Trends Ecol. Evol..

[34] Gerhardt KE, Huang X-D, Glick BR, Greenberg BM (2009). Phytoremediation and rhizoremediation of organic soil contaminants: potential and challenges. Plant Sci..

[35] Sage RF (2004). The evolution of C4 photosynthesis. New phytologist.

[36] Leme DM, Marin-Morales MA (2009). *Allium cepa* test in environmental monitoring: a review on its application. Mutat.Res.-Rev Mutat..

[37] Herrero O (2012). Toxicological evaluation of three contaminants of emerging concern by use of the *Allium cepa*test. Mutat. Res.- Genet.Tox. En.

[38] Leme DM, Marin-Morales MA (2009). *Allium cepa* test in environmental monitoring: A review on its application. Mutat. Res-Rev. Mutat..

[39] Rank J, Nielsen MH (1998). Genotoxicity testing of wastewater sludge using the *Allium cepa* anaphase-telophase chromosome aberration assay. Mutat Res.- Genet. Tox. En..

[40] Cabaravdic M (2010). Induction of chromosome aberrations in the *Allium cepa* test system caused by the exposure of cells to Benzo(*a*)pyrene. Med. Arch..

[41] Fiskesjo, G. *Allium* test for screening chemicals; evaluation of cytological parameters. *Plants for Environmental Studies*, 308–333 (1997).

[42] Liman R, Ciğerci İH, Öztürk NS (2015). Determination of genotoxic effects of Imazethapyr herbicide in Allium cepa root cells by mitotic activity, chromosome aberration, and comet assay. Pestic. Biochem. Phys..

[43] USEPA (2005). Test methods for evaluating solid waste, physical/chemical methods.

[44] Gee GW, Or D (2002). 2.4 Particle-size analysis. Methods of Soil Analysis.

[45] Grant, I. F. Soil Processes. Ecological Monitoring Methods for the Assessment of Pesticides Impact in the Tropics (Grant IF and Tingle, CCD Editors). *Natural Resources Institute, Chatham, UK*, 149–157 (2002).

[46] Sivaram AK, Logeshwaran P, Lockington R, Naidu R, Megharaj M (2018). Impact of plant photosystems in the remediation of benzo[a]pyrene and pyrene spiked soils. Chemosphere.

[47] Fu S, Cheng W, Susfalk R (2002). Rhizosphere respiration varies with plant species and phenology: a greenhouse pot experiment. Plant Soil..

[48] Robichaux RH, Pearcy RW (1980). Photosynthetic responses of C3 and C4 species from cool shaded habitats in Hawaii. Oecologia.

[49] Yamori W, Hikosaka K, Way DA (2014). Temperature response of photosynthesis in C3, C4, and CAM plants: temperature acclimation and temperature adaptation. Photosynth. Res..

[50] Parrish ZD (2006). Accumulation of weathered polycyclic aromatic hydrocarbons (PAHs) by plant and earthworm species. Chemosphere.

[51] Szmigielska AM, Van Rees KC, Cieslinski G, Huang P (1996). Low molecular weight dicarboxylic acids in rhizosphere soil of durum wheat. J. Agr. Food Chem..

[52] Casida L, Klein D, Santoro T (1964). Soil dehydrogenase activity. Soil Sci..

[53] Carter, M. R. *Soil sampling and methods of analysis*. (CRC Press, 1993).

[54] Huang X-D, El-Alawi Y, Penrose DM, Glick BR, Greenberg BM (2004). A multi-process phytoremediation system for removal of polycyclic aromatic hydrocarbons from contaminated soils. Environ.Pollut..

[55] Gao Y, Ling W, Wong MH (2006). Plant-accelerated dissipation of phenanthrene and pyrene from water in the presence of a nonionic-surfactant. Chemosphere.

[56] Jones KC, Alcock RE, Johnson D, Semple KT, Woolgar P (1996). Organic chemicals in contaminated land: analysis, significance and research priorities. Land Contamination and Reclamation.

[57] Oleszczuk P (2009). Application of three methods used for the evaluation of polycyclic aromatic hydrocarbons (PAHs) bioaccessibility for sewage sludge composting. Bioresource Technol..

[58] Pfosser M, Heberle‐Bors E, Amon A, Lelley T (1995). Evaluation of sensitivity of flow cytometry in detecting aneuploidy in wheat using disomic and ditelosomic wheat–rye addition lines. Cytometry.

[59] Yıldız M, Ciğerci İH, Konuk M, Fidan AF, Terzi H (2009). Determination of genotoxic effects of copper sulphate and cobalt chloride in *Allium cepa* root cells by chromosome aberration and comet assays. Chemosphere.

[60] Singh NP, McCoy MT, Tice RR, Schneider EL (1988). A simple technique for quantitation of low levels of DNA damage in individual cells. Exp. Cell Res..

