# Supplementary material for: Comparison of plants with C3 and C4 carbon fixation pathways for remediation of polycyclic aromatic hydrocarbon contaminated soils
Source: Sci Rep. 2018 Feb 1;8:2100. doi: 10.1038/s41598-018-20317-0 (PMC5794979; doi:10.1038/s41598-018-20317-0)
Supplement: Supplementary file 1 — Supplementary Information [file 41598_2018_20317_MOESM1_ESM.pdf]

Comparison of plants with C3 and C4 carbon fixation pathways for remediation of polycyclic aromatic hydrocarbon contaminated soils

Anithadevi Kenday Sivaram[1,2,3], Panneerselvan Logeshwaran [1,2,3], Suresh R Subashchandrabose[1,2,3], Robin Lockington[2,3] , Ravi Naidu[1,2,3], Mallavarapu Megharaj[1,2,3*]

[1]Global Centre for Environmental Remediation, Faculty of Science, The University of Newcastle (UoN), University Drive, Callaghan NSW 2308, Australia.

[2]Centre for Environmental Risk Assessment and Remediation (CERAR), University of South Australia, Mawson Lakes, SA, 5095, Australia.

[3]Cooperative Research Centre for Contamination Assessment and Remediation of the Environments, ATC Building, The University of Newcastle, University Drive, Callaghan, NSW 2308, Australia.

*Correspondence to: megh.mallavarapu@newcastle.edu.au

Anions	Concentration (mg kg^{-1})
Fluoride	1.3
Chloride	28.4
Bromide	< 0.5
Nitrate	9.8
Sulphate	70.6

Table S1. Concentrations of anions

Inorganics	Water soluble inorganics (mg kg^{-1})	Total inorganics (mg kg^{-1})
P	0.00	126.3
S	99.70	214.3
K	19.30	3113.4
Cr	0.01	13.6
Mn	*nd*	259.1
Ni	*nd*	6.4
Co	*nd*	11.2
Cu	0.01	15.6
Zn	*nd*	34.2
As	0.05	17.9
Cd	*nd*	0.1
Pb	*nd*	22.0

Table S2. Elemental concentrations

**(c) PAH**	**No. of benzene rings**	**Molecular weight**	**Soil concentration (mg kg^{-1})**
Naphthalene	2	128.8	8.0
Acenaphthylene	2	152.2	9.7
Acenaphthene	2	154.2	66.8
Fluorene	3	166.2	11.1
Phenanthrene	3	178.2	45.3
Anthracene	3	178.2	21.4
Fluoranthene	3	202.3	56.4
Pyrene	4	202.3	105.9
Benz[*a*]anthracene	4	228.3	31.4
Chrysene	4	228.3	76.9
Benzo[*b*]fluoranthene	4	252.3	77.0
Benzo[*k*]fluoranthene	4	252.3	52.5
Benzo[*a*]pyrene	5	252.3	108.8
Dibenz[*a,h*]anthracene	5	278.4	92.9
Benzo[*ghi*]perylene	6	276.3	56.8
Indeno(123*cd*)pyrene	5	276.3	172.2
**Total**			**995.1**

Table S3 Initial concentrations of 16 PAHs.

Common name	Scientific name	Type	Source
**C3 plants**			
Cowpea	*Vigna unguiculata*	Legume	William group, NSW
Sunflower	*Helianthus annuus*	Oilseed	Bunnings, SA
Wallaby grass	*Austrodanthonia caespitosa*	Australian native grass	Native seeds, VIC
**C4 plants**			
Maize	*Zea mays*	Crop	Pacific seeds, SA
Sudan grass	*Sorghum sudanense*	Pasture crop	Pacific seeds, SA
Vetiver	*Vetiveria zizanoides*	Grass	Local farmer, Adelaide Hills, SA

Table S4 Plants used

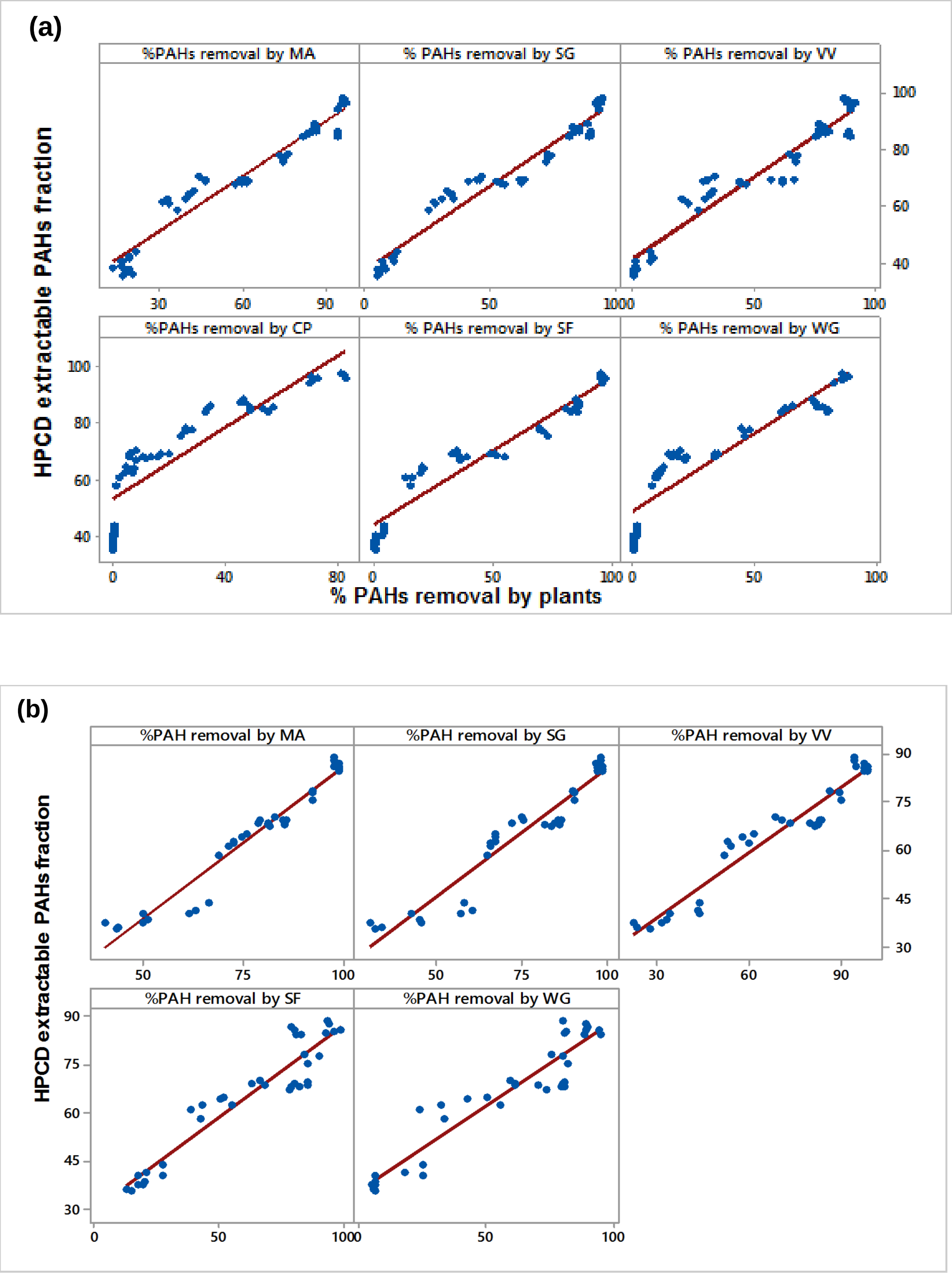

Figure S1 Relationship between HPCD extractable PAHs fraction and PAHs removal by C3 and C4 plants. **a)** after 60 days of plant growth **b)** After 120 days of plant growth MA - maize, SG - sudan grass, VV - vetiver, SF - sunflower and WG - wallaby grass.

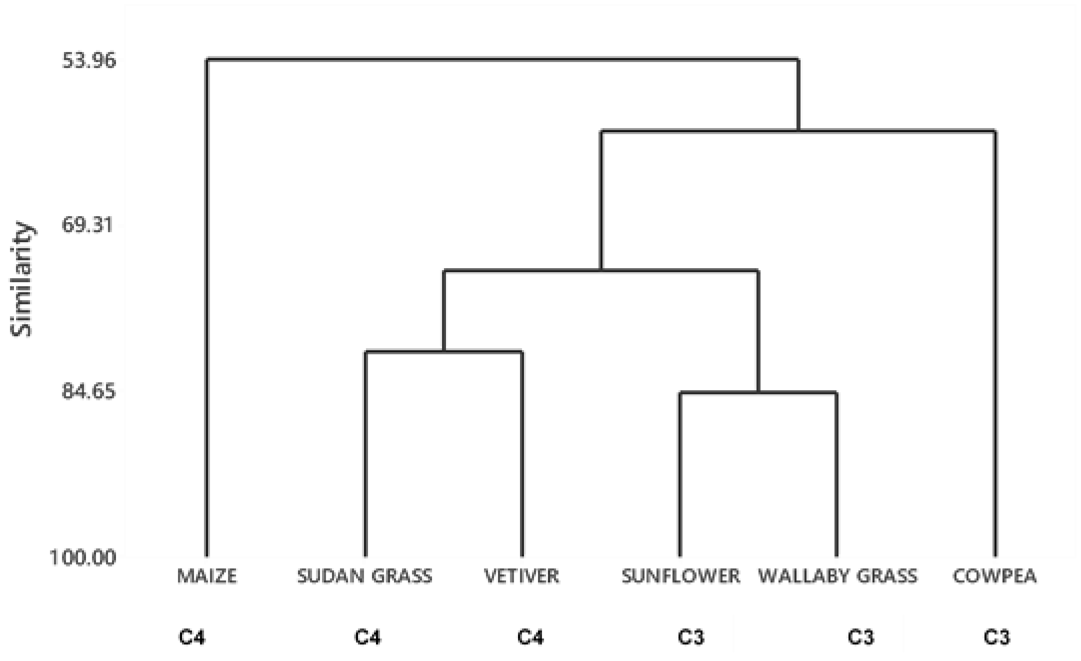

Figure S2 Dendrogram showing similarities/ differences between C3 and C4 plants used in terms of percentage of PAHs removed.
